# Thromboxane Modulates Endothelial Permeability

**DOI:** 10.1155/S0962935194000190

**Published:** 1994

**Authors:** J. M. Klausner, S. Abu-Abid, J. S. Alexander, R. Hanshke-Mineau, G. Goldman, N. Morel, C. R. Valeri, D. Shepro, H. B. Hechtman

**Affiliations:** 1 Department of Surgery Tel Aviv Sourasky Medical Center Tel Aviv University Israel; 2 Department of Surgery Brigham and Women's Hospital Harvard Medical School Boston MA USA; 3 Naval Blood Research Laboratory Boston University School of Medicine Boston MA USA; 4 Biological Science Center Boston University Boston MA USA

## Abstract

The study tests the role of thromboxane in modulating microvascular permeability *in vitro*. Cultured monolayers of bovine aortic endothelial cells were challenged with the thromboxane (Tx) mimic U46619. This led to disassembly of actin microfilaments, cell rounding, border retraction and interendotheHal gap formation. Pretreatment with the Tx receptor antagonist SQ 29,548 prevented the Tx mimic-induced cytoskeletal changes. The Tx mimic also altered endothelial cell barrier function. Increased permeability was indicated by the increased passage of labelled albumin across monolayers cultured on microcarriers, relative to untreated endothelial cells (p < 0.05). Furthermore, electron microscopy of endothelial cells cultured on the basement membrane of human placental amnion indicated increased permeability based on wide, interendotheHal gap formation and transit of the tracer horseradish peroxidase. Quantification of interendothelial gaps revealed an eleven-fold increase with the Tx mimic relative to untreated endothial cells (p < 0.05) and prevention by pretreatment with the Tx receptor antagonist (p < 0.05). These data indicate that Tx directly modulates the permeability of endothelial cell *in vitro*.


Mediators of Inflammation 3, 149–153 (1994)

### Thromboxane modulates endothelial permeability

J. M. Klausner,[1,2,CA] S. Abu-Abid,[1]

J. S. Alexander,[4] R. Hanshke-Mineau,[4]

G. Goldman,[2] N. Morel,[4] C. R. Valeri,[3]

D. Shepro,[4] and H. B. Hechtman[2]

Department of Surgery, Tel Aviv Sourasky Medical Center, Tel Aviv University, Israel; [2]Department of Surgery, Brigham and Women's Hospital, Harvard Medical School; [3]Naval Blood Research Laboratory, Boston University School of Medicine; and [4]Biological Science Center, Boston University, Boston, MA, USA

[CA] Corresponding Author

**This study tests the role of thromboxane in modulating microvascular permeability *in vitro*. Cultured monolayers of bovine aortic endothelial cells were challenged with the thromboxane (Tx) mimic U46619. This led to disassembly of actin microfilaments, cell rounding, border retraction and interendothelial gap formation. Pretreatment with the Tx receptor antagonist SQ 29,548 prevented the Tx mimic-induced cytoskeletal changes. The Tx mimic also altered endothelial cell barrier function. Increased permeability was indicated by the increased passage of labelled albumin across monolayers cultured on microcarriers, relative to untreated endothelial cells ($p < 0.05$). Furthermore, electron microscopy of endothelial cells cultured on the basement membrane of human placental amnion indicated increased permeability based on wide, interendothelial gap formation and transit of the tracer horseradish peroxidase. Quantification of interendothelial gaps revealed an eleven-fold increase with the Tx mimic relative to untreated endothial cells ($p < 0.05$) and prevention by pretreatment with the Tx receptor antagonist ($p < 0.05$). These data indicate that Tx directly modulates the permeability of endothelial cells *in vitro*.**

**Key words:** Cytoskeleton, Endothelium, Permeability, Thromboxane

### Introduction

Thromboxane (Tx)$A_2$, a potent vasoconstrictor agent is thought to be a significant mediator of the respiratory failure noted in a variety of clinical and experimental disorders including those related to ischaemia and reperfusion, endotoxaemia, complement activation, microembolization, aspiration and burns.[1,2] Since $TxA_2$ has a 30 s half-life, interpretation of its pathophysiological role has been derived from measurement of its relatively stable hydrolysis product, $TxB_2$, and from effects observed when Tx synthesis was blocked pharmacologically.[2] Conclusions drawn from these observations are open to potential error. The presence of increased $TxB_2$ levels in plasma does not necessarily reflect the involvement of $TxA_2$ in organ pathology. Further, many Tx inhibitors, such as drugs blocking cyclooxygenase are nonselective and may inhibit the synthesis, binding or degradation of other arachidonic acid (AA) metabolites and possibly oxygen free radicals.[2] Even relatively selective inhibitors of Tx synthetase may redirect the endoperoxide precursors away from the $TxA_2$ pathway towards prostaglandin (PG) or leukotriene synthesis.[2] Finally, studies designed to test whether a pathophysiological effect could be induced by infusion of an agonist have also been marred by the use of nonspecific agents such as AA.[3-6] Infusion of this precursor can result in the production of a variety of cyclooxygenase as well as lipoxygenase products. It is therefore not surprising that conflicting results have been reported regarding the effects of Tx on microvascular permeability.[3-6]

Significant evidence has been obtained indicating that $TxA_2$ may produce vasoconstriction and bronchospasm and that Tx can induce platelet aggregation and leukocyte adhesion.[2] However, given the methodological problems, the role of Tx in moderating microvascular increased permeability is less certain.[2-6] This study was designed to test the putative Tx action on the vascular barrier utilizing a stable Tx mimic and a Tx receptor antagonist.

### Materials and Methods

*Studies of barrier function:* The effect of the Tx mimic on endothelial cell (EC) barrier function was evaluated using the permeability assay originally described by Boiadjieva *et al.*[7] and modified by Bottaro *et al.*[8] This technique allows the quantitation of transendothelial solute transport by measuring the movement of tracer dye bound to albumin through a monolayer of endothelial cells grown on microcarrier beads. The movement of the tracer dye from the medium to the bead matrix is followed spectrophotometrically.

Bovine aortic EC were obtained as described previously.[8-10] Endothelial cells were identified by

© 1994 Rapid Communications of Oxford Ltd their characteristic growth morphology, colony pattern and presence of factor VIII antigen.[8,11] Primary cultures were subcultured a maximum of two times before seeding onto Cytodex 3 microcarrier beads (Pharmacia, Inc., Piscataway, NJ). Microcarriers were kept in suspension using a Techne MCS-104S magnetic stirrer (Techne Inc., Needham, MA) operated at 44 rpm inside a conventional tissue culture incubator. Endothelial cells were seeded onto the microcarriers at a minimum density of 10 cells per bead, and allowed to grow for at least 6 days post-confluency.

Tryplan blue (TB) dye (Fisher Scientific, Pittsburg, PA) and bovine serum albumin (BSA) (Sigma, St. Louis, MO) were added to DMEM so that the final concentrations of TB and BSA were 0.2% and 0.45% respectively. The molecular weight of this TB–BSA complex was 100 000.[8] Five ml samples of microcarrier suspension, with a concentration of 40 000 microcarrier beads/ml were placed in a plastic vial. The growth medium was replaced by DMEM plus the TB-BSA dye solution to which the Tx mimic U46619 (9,11-dideoxy-M, 9-epoxymethano-$PGE_2$) (Upjohn, Kalamazoo, MI) at $10^{-4}$ M or $10^{-5}$ M or the Tx receptor antagonist at $10^{-4}$ M, or both mimic and antagonist at $10^{-4}$ M respectively had been added. Five sets of experiments were performed. Non-cell coated microcarriers (naked beads) were included in every experiment to provide a maximum for the rate of TB–BSA uptake into the microcarrier matrix. The vehicles of either Tx mimic or the Tx receptor antagonist SQ 29,548 (Squibb, Princeton, NJ) were also added in two experiments. The vials were placed in a 37°C water bath and agitated gently. At 5 and 15 min 150 μl of beads and dye solution were removed from the vials and placed on an oil cushion of dibutyl:dioctylphthalate in a ratio of 3:1. The aliquots were then centrifuged for 30 s at 1500 × **g**. This effectively separated the microcarriers from the medium and terminated the dye uptake by the microcarrier beads. Dye concentration in the supernatant was then assayed by mixing a 50 μl aliquot of supernatant with 950 μl of distilled water. Absorbance was read at 580 nm with a Beckman DU-50 spectrophotometer. All samples were analysed in triplicate. Results are expressed as the reciprocal of the supernatant absorbance.[8]

*Endothelial cell morphology and cytoskeleton:* EC were seeded onto 1.2 cm, 1.5% gelatin coated coverslips. At 3 days post confluency, cell monolayers were exposed to the Tx mimic or Tx receptor antagonist or their combination in DMEM at concentrations of $10^{-4}$ to $10^{-5}$ M for up to 4 h. At 5 min following incubation, monolayers were washed with phosphate buffered formaldehyde (Fisher Scientific) for 15 min at 25°C. Monolayers were washed free of fixative and permeabilized using Triton X-100 detergent (Fisher Scientific) in PBS for 5 min. Endothelial cell cytoskeleton was visualized by staining with the F-actin fluorescent stain rhodamine–phalloidin[10] (Molecular Probes, Junction City, OR), 1 U/coverslip for 30 min, washed 3× in PBS and mounted and sealed in PBS/glycerol (1:1). Cells were illuminated for fluorescent photomicrography using a Zeiss universal microscope. Three sets of experiments were performed.

*Electron microscopy:* Post-confluent EC cultured on the basement membrane of human placental amnion[12] were treated with the Tx mimic or the Tx receptor antagonist or their combination at concentrations of $10^{-5}$ M respectively in DMEM for 5 min at 37°C. After brief rinsing of the EC, the tracer horseradish peroxidase (HRP) (Sigma) 0.5 mg/ml was added for 10 min at 37°C. Monolayers were then fixed and processed for HRP localization.[13] Thin 90 mm sections were examined on a Phillips 410 electron microscope. Thick 2.5 mm sections were mounted on glass slides and viewed under an in-

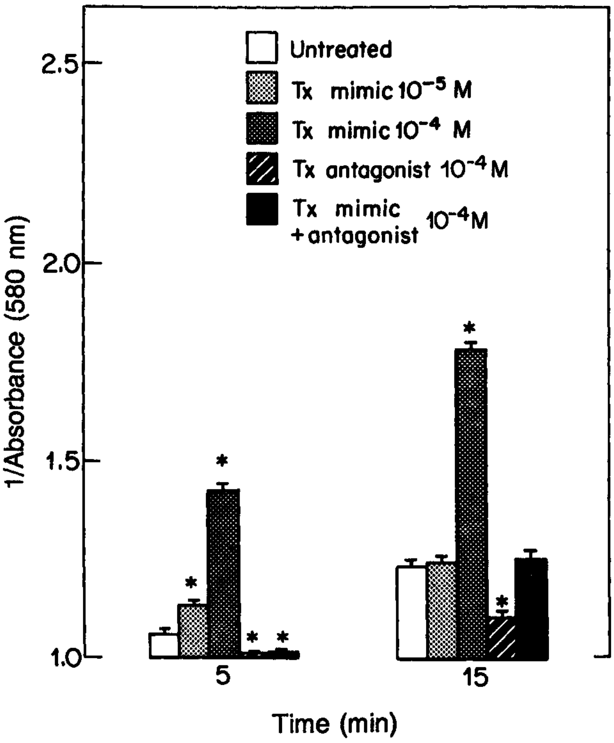

FIG. 1. Dye–albumin concentration in the supernatant varied inversely with the passage of the dye–albumin conjugate across the endothelial cell (EC) monolayer grown on the microcarrier beads. Untreated EC had a measurable barrier and decreased the passage of albumin relative to naked beads. Incubation with the Tx mimic increased the passage of dye labelled albumin across the EC monolayer indicating decreased barrier function. Pretreatment with the Tx receptor antagonist SQ 29,548 prevented the Tx induced increase in permeability. Treatment of unstimulated EC with the Tx antagonist alone decreased the passage of albumin relative to untreated EC indicating enhanced barrier function. Asterisks refer to significance relative to untreated EC, and daggers indicate significant differences between EC treated with Tx mimic alone and the combination of Tx mimic and antagonist. □, untreated; ▨, Tx mimic ($10^{-5}$ M); ■, Tx mimic ($10^{-4}$ M); ▨, Tx antagonist ($10^{-4}$ M); ■, Tx mimic + antagonist (both at $10^{-4}$ M).

verted Zeiss microscope (× 400). Changes in permeability were assessed by following the passage of HRP through the EC monolayer. In addition, the number of interendothelial leakage sites and enlarged gaps were counted in ten randomly selected microscopic fields.

Results are expressed in the text and figures as mean ± standard error. Differences between means were tested by an analysis of variance, paired and non-paired *t*-test. When multiple time points were compared the Bonferroni test was applied.[14] Significance was accepted if $p < 0.05$.

### Results

Bovine aortic EC grown to confluence on microcarrier beads formed a measurable barrier to

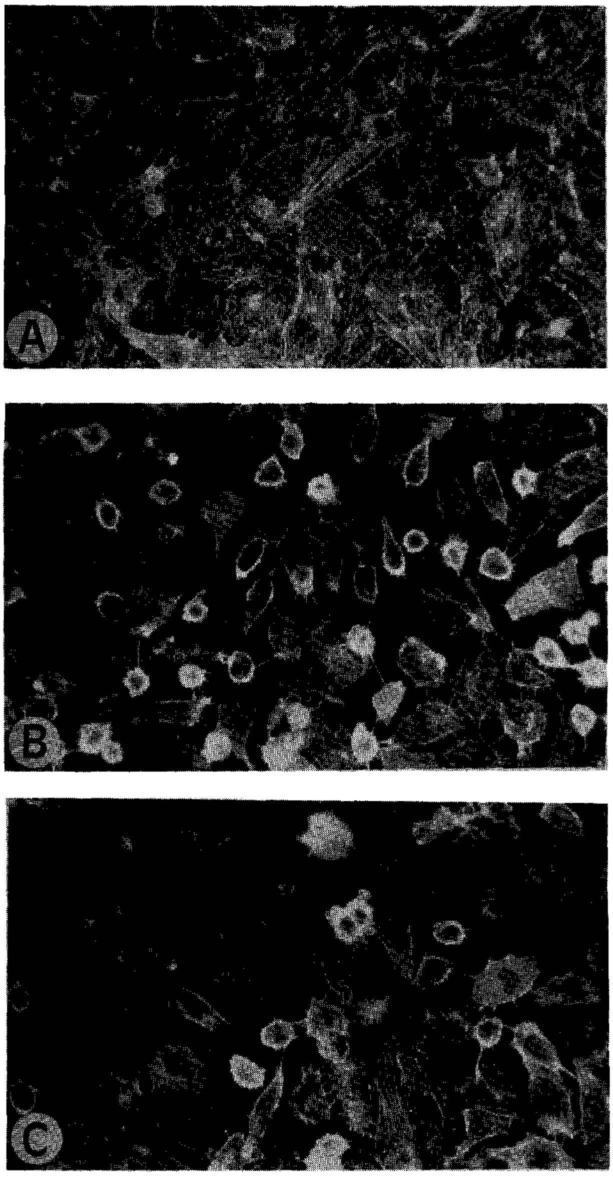

FIG. 2. Compared to untreated EC (A), in which the actin microfilament elements of the cytoskeleton are apparent, cells incubated for 5 min with Tx mimic at $10^{-4}$ M (B) or at $10^{-5}$ M (C) showed disassembly of actin microfilaments, border retraction, cell rounding and interendothelial gap formation (× 1150).

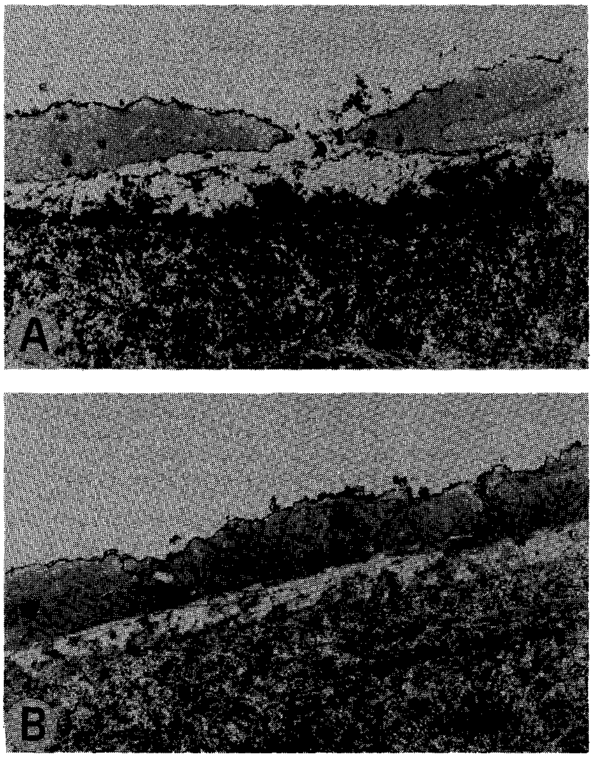

FIG. 3. The electron micrograph (× 5400) depicts a large interendothelial gap junction, 2.3 in width in a representative field of $10^{-5}$ M Tx mimic treated EC monolayer grown on amnion (A). The HRP tracer is seen in the gap and underneath the monolayer (arrows). Untreated EC appear with tight interendothelial junctions (B).

the passage of the TB–BSA conjugate into the microcarrier matrix and the amount of TB–BSA taken up by the EC covered microcarriers was significantly less than the amount taken up by 'naked' microcarriers (Fig. 1). Treatment of EC with the Tx mimic at concentrations of $10^{-4}$ M and $10^{-5}$ M increased TB–BSA passage across the EC into the microcarrier matrix after 5 and 15 min, relative to untreated cells ($p < 0.01$) (Fig. 1). This effect was more pronounced for the higher concentration of $10^{-4}$ M. The Tx antagonist SQ 29,548 enhanced the barrier function as indicated by reduced passage of the tracer relative to untreated EC ($p < 0.01$) (Fig. 1). Incubation of EC with SQ 29,548 $10^{-4}$ M for 5 min prior to their exposure to Tx mimic at $10^{-4}$ M prevented the increased TB–BSA passage across the EC induced by Tx mimic alone (Fig. 1). The vehicles of either Tx mimic or SQ 29,548 did not affect barrier function relative to untreated EC (data not shown).

Compared with untreated EC, cells challenged with the Tx mimic for 5 min showed border retraction, cell rounding, disassembly of EC actin microfilaments and interendothelial gap formation (Fig. 2). These changes were not quantified but were more pronounced with Tx mimic at $10^{-4}$ M compared to a concentration of $10^{-5}$ M. The latter however induced cytoskeletal changes that were apparent relative to untreated EC. Pretreatment with SQ 29,548

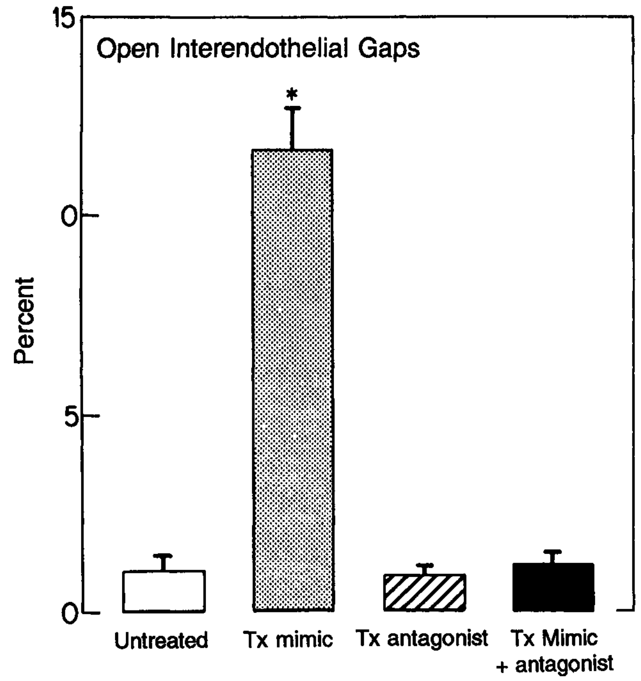

FIG. 4. Tx mimic, $10^{-5}$ M led to an 11.4-fold increase in the number of open interendothelial gap junctions relative to untreated EC. Pretreatment with the Tx receptor antagonist. ($10^{-6}$ M) prevented Tx induced gap formation. Asterisk indicates significance relative to untreated EC.

prevented the Tx mimic induced cytoskeletal changes.

Electron microscopy indicated increased permeability based on wide interendothelial gap formation and the transit of HRP (Fig. 3). EC monolayers in thin sections treated with the Tx mimic at a concentration of $10^{-5}$ M for 5 min revealed 287 leakage sites per 576 cells (50%), compared with 194 leakage sites per 925 cells (21%) for untreated EC ($p < 0.05$). The Tx mimic treated EC monolayers in thick sections showed large interendothelial gaps (Fig. 3) Quantification of interendothelial gaps in these sections revealed an 11.1-fold increase in the number of open gaps in EC treated with $10^{-5}$ M Tx mimic relative to untreated EC ($p < 0.05$) (Fig. 4). The Tx mimic induced gap formation was prevented by pretreatment with the Tx receptor antagonist (Fig. 4).

### Discussion

The chemically stable endoperoxide analogue used in these studies is believed to act as a biological mimic of $TxA_2$. This is indicated by its ability to constrict smooth muscle and, in higher dosage, to aggregate platelets and increase white blood cell chemotaxis.[1,2,15,16] Further, U46619 and $TxA_2$ display the same pattern of agonist responses on lung strips and vascular preparations from several species.[1,2] Finally, the ability of the Tx receptor antagonist to inhibit the effects of native $TxA_2$ as well as the effects of the mimic[1,2,16] indicate identical receptor sites and actions for native $TxA_2$ and the Tx mimic.

The permeability changes required Tx mimic concentrations that were very high compared to other *in vitro* systems. Nevertheless, the suppression by the Tx receptor antagonist makes a nonspecific effect on endothelial cytoskeleton and permeability rather unlikely.

We believe that Tx acts to moderate microvascular permeability by altering the EC cytoskeleton. Certain cytoskeletal elements, especially actin micro-filaments, appear to regulate EC mobility, structural relationship to adjacent cells and therefore interendothelial junctions and barrier function.[10,17–20] Agents such as phalloidin promote microfilament assembly, thereby tightening interend-othelial junctions and enhancing barrier function.[21] Similar to native $TxA_2$, the Tx mimic as well as other permeability promoting agents such as histamine, lead to a reversible disassembly of cytoskeletal actin microfilaments, events associated with widening of interendothelial junctions and increased permeability to protein.[10,17,19,20] Despite the uniformity of these observations, *in vivo* studies by others have failed to demonstrate increased lung permeability with agents such as histamine, arachidonic acid metabolites, and platelet activating factor, despite the ability of these agents to affect EC cytoskeleton *in vitro*.[6,22,23]

The application of aortic EC cultures as models of *in vivo* lung permeability is limited and interpretations must be made cautiously. Our results do not preclude the possibility that Tx may affect aortic EC cytoskeleton and barrier function differently than pulmonary microvessels. In fact, Maron's[22] observation suggests that intrinsic differences in the regulation of microvascular permeability exist between the systemic and pulmonary vasculature; histamine induced increased permeability of canine systemic blood vessels but failed to affect the barrier function in the dog and rabbit lung.[22] However, other studies indicate that histamine[24] as well as other agents, that is leukotrienes, interleukin-1 and interleukin-2 and platelet activating factor, increase the permeability of both the systemic and the pulmonary vasculature.[17,18,23,25] These conflicting results may relate to different methodologies and species. For example, the sheep lung may be especially susceptible to changes in permeability because of its rich population of intravascular macrophages.[23,26] These cells can respond to a permeability promoting agent by further release of inflammatory mediators that may themselves affect the vascular integrity in the lung.[26] Despite these considerations, aortic EC and pulmonary microvessel EC have been found to be quantitatively comparable with regard to the passage of albumin[9] suggesting that data from aortic EC may be extrapolated to pulmonary EC.

Thromboxane $A_2$ is thought to have additional indirect permeability effects via its interaction with circulating WBC and perhaps macrophages. Thus,

$TxA_2$ can enhance neutrophil endothelial adhesion[27] and promote neutrophil diapedesis through EC monolayers.[15] The Tx mimic has identical actions on WBC–EC interaction when tested *in vitro*.[15] Further, infusion of the Tx mimic in swine led to neutrophil sequestration in the lungs.[28] Activation of these sequestered neutrophils may lead to increased permeability. Although neutrophils may enhance Tx induced permeability they are not necessary for its expression. In other studies oedema has been reported in isolated lungs, free of white blood cells, challenged with nicotine[29] or alphathrombin.[30] These agents stimulate Tx synthesis and lead to Tx dependent increases in permeability.

Concentrations of $TxB_2$ are elevated in a number of clinical and experimental settings of increased permeability. This does not document a casual relationship. Thus, the increased plasma Tx concentrations during endotoxaemia are not as important as other mediators of permeability since Tx inhibition is not preventative.[1,31] On the other hand, in other settings Tx appears central in mediating microvascular permeability. For example, inhibition of Tx prevents increased microvascular permeability in the ischaemic reperfused dog hind limb;[32] in sheep lungs following microembolization,[30] or lower torso ischaemia with reperfusion;[33] and in rat lungs challenged with inhaled nicotine.[29]

In summary, these data suggest that thromboxane $A_2$ directly moderates microvascular permeability *in vitro*.

### References

1. Halushka PB, Lefer AM. Thromboxane $A_2$ in health and disease. *Fed Proc* 1987; **46**: 131–132.
2. Ogletree ML. Overview of physiological and pathophysiological effects of thromboxane $A_2$ *Fed Proc* 1987; **46**: 133–138.
3. De Clerck F, Loots W, Somers Y, Van Gorp L, Verheyen A, Wouters L. Thromboxane $A_2$-induced vascular endothelial cell damage and respiratory smooth muscle contraction: inhibition by flumarizine, a $Ca^{2+}$-overload blocker. *Arch Int Pharmacodyn* 1985; **274**: 4–23.
4. Seeger W, Walmrath D, Menger M, Neuhof H. Increased lung vascular permeability after arachidonic acid and hydrostatic challenge. *J Appl Physiol* 1986; **61**: 1781–1789.
5. Selig WM, Noonan TC, Kern DF, Malik AB. Pulmonary microvascular responses to arachidonic acid in isolated perfused guinea pig lung. *J Appl Physiol* 1986; **60**: 1972–1979.
6. Townsley MI, Korthuis RJ, Taylor AE. Effects of arachidonate on permeability and resistance distribution in canine lungs. *J Appl Physiol* 1985; **58**: 206–210.
7. Boiadjieva S, Hallberg C, Hogstorm M, Busch C. Exclusion of trypan blue from microcarriers by endothelial cells. An *in vitro* barrier function test. *Lab Invest* 1984; **50**: 239–246.
8. Bottaro D, Shepro D, Peterson S, Hechtman HB. Serotonin, norepinephrine and histamine mediation of endothelial cell barrier function *in vitro*. *J Cell Physiol* 1986; **128**: 189–194.
9. Bottaro D, Shepro D, Hechtman HB. Heterogeneity of intimal and microvessel endothelial cell barriers *in vitro*. *Microvasc Res* 1986; **32**: 389–398.
10. Welles SL, Shepro D, Hechtman HB. Vasoactive amines modulate actin cables (stress fibers) and surface area in cultured bovine endothelium. *J Cell Physiol* 1985; **123**: 337–342.
11. Jaffe EA, Nachman RL, Becker CG, Mimick CR. Culture of human endothelial cell derived from umbilical veins; identification by morphologic and immunologic criteria. *J Clin Invest* 1973; **52**: 2745–2756.
12. Liotta LA, Lee CW, Morakis DJ. New method for preparing large surface of intact human basement membrane for tumor invasion studies. *Cancer Let* 1980; **11**: 141–152.
13. Wolosewick JJ, Porter KR. Preparation of cultured ells for electron microscopy. In: Maramorosch K, Hirumi K, eds. *Practical Tissue Culture Application*. New York: Academic Press, 1979; 59–85.
14. Wallenstein S, Zucker CL, Fleiss JL. Some statistical methods useful in circulation research. *Circ Res* 1980; **47**: 1–9.
15. Doukas J, Hechtman HB, Shepro D. Endothelial-secreted arachidonic acid metabolites modulate polymorphonuclear leukocyte chemotaxis and diapedesis *in vitro*. *Blood* 1988; **71**: 771–779.
16. Lefer AM, Darius H. A pharmacological approach to thromboxane receptor antagonism. *Fed Proc* 1987, **46**: 144–148.
17. Del Vecchio PJ, Silfinger-Birnboim A, Shepard JM, Bizios R, Cooper JA, Malik AB. Endothelial monolayer permeability to macromolecules. *Fed Proc* 1987; **37**: 647–657.
18. Meyrick B, Perkett EA, Harris TR, Brigham KL. Correlation of permeability with the structure of the endothelial layer of pulmonary artery intimal explants. *Fed Proc* 1987; **46**: 2516–2520.
19. Shasby DM, Shasbi SS, Sullivan JM, Peach MJ. Role of endothelial cell cytoskeleton in control of endothelial permeability. *Circ Res* 1982; **51**: 657–661.
20. Welles SL, Shepro D, Hechtman HB. Eicosanoid modulation of stress fibers in cultured bovine aortic endothelial cells. *Inflammation* 1985; **9**: 439–450.
21. Alexander JS, Shepro D, Hechtman HB. Phalloidin improves endothelial barrier function and reduces inflammatory permeability *in vitro*. *Microvasc Res* 1988; **35**: 308–315.
22. Maron MB. Differential effects of histamine on protein permeability in the dog lung and forelimb. *Am J Physiol* 1982; **242**: H565–H572.
23. Newman JH. Lung vascular injury. *Chest* 1988; **93S**: 139–146.
24. Brigham KL, Owen PJ. Increased sheep lung vascular permeability caused by histamine. *Circ Res* 1975; **37**: 647–657.
25. Burhop KE, Garcia JGN, Selig WM, *et al.* Platelet-activating factor increases lung vascular permeability to protein. *J Appl Physiol* 1986. **61**: 2210–2217.
26. Staub NC. Pulmonary intravascular macrophages. *Chest* 1988; **93S**: 84–85.
27. Spagnuolo PJ. Ellner JJ, Hassid A, Dunn MJ. Thromboxane $A_2$ mediates augmented polymorphonuclear leukocyte adhesiveness. *J Clin Invest* 1980; **66**: 406–414.
28. Slotman GJ, Teplitz C, Yellin SA, Farruyir R. Interaction of thromboxane $A_2$ and tissue pathology during graded bacteremia. *J Trauma* 1987; **27**: 167–175.
29. Lelcuk S, Threfall L, Valeri CR, Shepro D, Hechtman HB. Nicotine stimulates pulmonary parenchymal thromboxane synthesis. *Surgery* 1986; **100**: 836–840.
30. Horgan MJ, Fenton JN,II, Malik AB, Thrombin induced pulmonary vasoconstriction. *J Appl Physiol* 1987; **63**: 1993–2000.
31. Snapper JR, Hutchinson AA, Ogletree ML, Brigham KL. Effects of cyclooxygenase inhibitors on the alteration in lung mechanics caused by endotoxemia in the unanesthetized sheep. *J Clin Invest* 1983; **72**: 63–76.
32. Lelcuk S, Alexander F, Valeri CR, Shepro D, Hechtman HB. Thromboxane $A_2$ moderates permeability after limb ischemia. *Ann Surg* 1985; **200**: 642–648.
33. Klausner JM, Paterson IS, Kobzik L, Valeri CR, Shepro D, Hechtman HB. Thromboxane mediates limb ischemia induced pulmonary permiability. *Circ Res* 1989; **64**: 1178–1179.

ACKNOWLEDGEMENTS. This work was supported in part by The National Institute of Health, Grants No. GM24891-10, GM35141-03, HL16714-13; The U.S. Navy Office of Naval Research, Contract No. N00014-79-C-0168; The Brigham Surgical Group, Inc. and The Trauma Research Foundation.

**Received 13 December 1993; accepted in revised form 14 January 1994**
